# Supplementary material for: True amplification of spin waves in magnonic nano-waveguides
Source: Nat Commun. 2024 Feb 20;15:1560. doi: 10.1038/s41467-024-45783-1 (PMC10879122; doi:10.1038/s41467-024-45783-1)
Supplement: Supplementary file 1 — Supplementary Information [file 41467_2024_45783_MOESM1_ESM.pdf]

True amplification of spin waves in magnonic nano-waveguides

Supplementary information

Supplementary Note 1

The nonlinear frequency shift is a change in the frequency of magnetization precession due to a decrease in the static component of magnetization M_{ST} with increasing precession angle. The simplest way to understand the effects of anisotropy and angle of the static magnetic field on nonlinear frequency shift is to consider the frequency of the ferromagnetic resonance (FMR) for two limiting cases: in-plane (IP) magnetized film ($\theta = 0$) and out-of-plane (OP) magnetized film ($\theta = 90°$). In the first case, the FMR frequency can be expressed as $\omega_{\mathrm{IP}} = \gamma\sqrt{H_0(H_0 + 4\pi M_{\mathrm{eff}})}$. In the second case, it is $\omega_{\mathrm{OP}} = \gamma(H_0 - 4\pi M_{\mathrm{eff}})$. Here γ is the gyromagnetic ratio and M_{eff} is the effective magnetization. In films with perpendicular magnetic anisotropy[1], $4\pi M_{\mathrm{eff}} = 4\pi M_{\mathrm{ST}} - H_{\mathrm{a}} = (4\pi - N^{\mathrm{a}})M_{\mathrm{ST}}$, where M_{ST} is the static component of the magnetization, $H_{\mathrm{a}} = N^{a} M_{\mathrm{ST}}$ is the effective field of the anisotropy, and N^{a} is the anisotropy constant.

In the case, if there is no anisotropy or the effective field of the anisotropy H_{a} is smaller than $4\pi M_{\mathrm{ST}}$, $4\pi M_{\mathrm{eff}}$ is positive. Under these conditions, a decrease in M_{ST} leads to a decrease in ω_{IP} (negative nonlinear frequency shift) and an increase in ω_{OP} (positive nonlinear frequency shift).

In the case $H_{\mathrm{a}} > 4\pi M_{\mathrm{ST}}$, which is the case in our experiments, $4\pi M_{\mathrm{eff}}$ is negative. Under these conditions, a decrease in M_{ST} leads to an increase in ω_{IP} (positive nonlinear frequency shift) and a decrease of ω_{OP} (negative nonlinear frequency shift). Correspondingly, by changing the angle of the static magnetic field θ from 0 to 90 degrees, one can change the sign of the nonlinear frequency shift. Moreover, for a certain angle θ, one can achieve a situation where the nonlinear frequency shift vanishes.

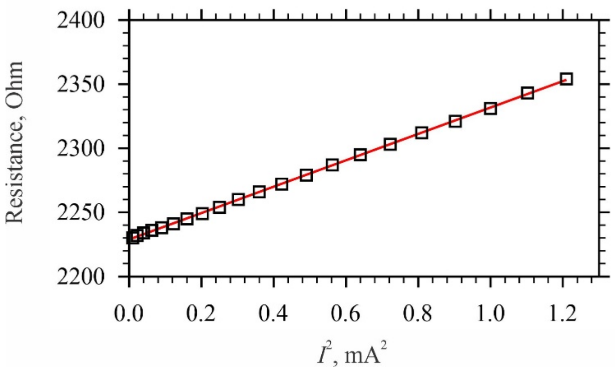

Supplementary Figure 1. Current dependence of the electrical resistance of the sample. Symbols show the experimental data obtained in the continuous-current regime. Line shows the linear fit. The intercept is 2229 Ohm, the slope is 103 Ohm mA^{-2}

Supplementary References

1. Kalinikos, B. A., Kostylev, M. P., Kozhus', N. V., & Slavin, A. N., The dipole-exchange spin wave spectrum for anisotropic ferromagnetic films with mixed exchange boundary conditions, *J. Phys.: Condens. Matter* **2**, 9861 (1990).
